# Development and validation of makeup and sexualized clothing questionnaires

**DOI:** 10.1186/s40337-017-0171-1

**Published:** 2017-11-22

**Authors:** Haylie Smith, Marisol Perez, Michael R. Sladek, Carolyn Black Becker, Tara K. Ohrt, Amanda B. Bruening

**Affiliations:** 1 Psychology Department Arizona State University, 950 South McAllister Avenue Room 237, Tempe, AZ 85287-1104 USA; 2 Department of Psychology, Trinity University, Center for the Sciences and Innovation room 253, One Trinity Place, San Antonio, TX 78212 USA

**Keywords:** Makeup, Sexualized clothing, Self-objectification, Pressure, College women

## Abstract

**Background:**

Body acceptance programs on college campuses indicated that collegiate women often report feeling pressure to dress in a sexualized manner, and use makeup to enhance beauty. Currently, no quantitative measures exist to assess attitudes and daily behaviors that may arise in response to perceived pressure to wear makeup or dress in a provocative manner. The goal of the current studies was to develop brief self-report questionnaires aimed at assessing makeup and sexualized clothing use and attitudes in young women.

**Methods:**

An exploratory factor analysis in a sample of 403 undergraduate women was used in Study 1 to create items to measure the pressure women feel to wear makeup and sexualized clothing. A confirmatory factor analysis (*N* = 153) was used in Study 2 to confirm the factor structure found in Study 1. An incremental validity analysis was also conducted in Study 2. Across both studies, participants completed online questionnaires.

**Results:**

In Study 1, items were developed for two questionnaires to assess perceived pressure to wear makeup and discomfort when not wearing makeup, and perceived pressure to wear sexualized clothing, and body image concerns with regards to sexualized clothing. The exploratory factor analyses revealed Unconfident and Unease scales for the Makeup Questionnaire (MUQ) and Body Dissatisfaction and Pressure scales for the Sexualized Clothing Questionnaire (SCQ). In Study 2, the confirmatory factor analyses confirmed the factor structure for the MUQ and SCQ. The incremental validity analysis revealed that these measures can be used to predict self-objectification and shape and weight concern in women.

**Conclusion:**

These studies provide preliminary support for the factor structure of two novel questionnaires aimed at assessing perceived pressure to wear makeup and sexualized clothing.

## Plain English summary

Women are increasingly promoted as objects in today’s society, especially in the media. As a result of this objectification of women, women feel the need to engage in daily behaviors, such as putting on makeup and sexualized clothing, to meet societies’ standards of beauty. Therefore, this study created scales to measure the pressure women feel to wear makeup and sexualized clothing. The scales for makeup assess women’s confidence without makeup, as well as their pressure to wear makeup all the time, even when running errands. The scales for sexualized clothing assess whether women avoid wearing sexualized clothing due to body image concerns, as well as the pressure they feel from society to wear revealing clothes. Then, we found that these scales can be used to predict harmful behaviors in women, such as seeing themselves as an object and being preoccupied with their shape and weight.

## Background

### Development and validation of makeup and sexualized clothing questionnaires

Since the 1980s, researchers note that certain environments within Western society treat women as sexual objects, with a focus on beauty and appearance [1–3]. Furthermore, Grauf, Murnen, & Krause note the increasingly sexualized depiction of girls in magazines over time demonstrates Western society’s escalating view of girls and women as sexual objects [4]. Self-objectification theory, which has been used to examine the consequences of treating women and girls as sexual objects (e.g., eating disorders, low self-esteem, and depression), proposes that a woman views herself as an object from a third-person perspective so as to be able to anticipate societal responses to her [5–7]. Research suggests that self-objectification can be associated with increased body monitoring, body shame, and appearance anxiety [5, 8]. Additionally, increased self-objectification is correlated with eating disorder pathology, body dissatisfaction, thin-ideal internalization, depression, and overall decreases in life satisfaction [9, 10]. In addition to its impact on mental health, self-objectification has been found to hinder performance on cognitive tasks [11, 12]. It is important to note, however, that the majority of research on self-objectification has focused on trait-level constructs (i.e., habitual patterns of behavior, attitudes and emotion); to date limited research has examined the daily impact of objectification.

As noted above, self-objectification may serve an adaptive function by allowing women to predict how others in an objectifying society will respond to them [5]. Taking a third party gaze, however, also may motivate women to engage in behaviors that allow them to conform with societal ideals, thereby increasing societal approval. For instance, the media, which commonly objectifies women, typically presents the ideal woman as wearing both makeup and sexualized clothing [13]. Theoretically, women who are higher in self-objectification should experience greater pressure to conform to societal appearance standards, which promote the use of sexualized clothing and makeup [5, 10].

Media campaigns promote makeup as a means to increase female beauty and attractiveness by enhancing some facial features and hiding others. As a result of successful media campaigns, U.S. consumers (mostly female consumers) spend billions of dollars on cosmetics each year [14, 15]. Self-objectification has been shown to predict momentary changes in women’s behaviors and performance (e.g., cognitive performance on the Stroop task), as well as more chronic changes in mood and behavior (e.g., depression, anxiety, eating disorders) [5]. Self-objectification also can be used to provide a framework for understanding women’s makeup use. For example, it is possible that women experience appearance anxiety due to the flaws they perceive on their faces. To reduce such appearance anxiety, women may wear makeup in an attempt to cover up their flaws or enhance their self-perceived attributes. To date, most makeup research focuses on how other people view women wearing makeup; for instance, one study found that men and women associate more implicit, positive attributes towards women wearing makeup [16]. More specifically, women wearing makeup were more likely to be viewed as high status professionals and as having positive personality traits compared to women not wearing facial makeup [16]. However, this study on makeup did not assess how women themselves felt about wearing makeup.

Images of women and girls in the media also link beauty with sexuality [4]. The sexualization of women permeates multiple facets of entertainment including television, billboards, magazines, video games, Facebook, and clothing stores [6, 13, 17, 18]. For example, stiletto heels were originally almost exclusively worn by porn stars and mainly depicted in pornography, but now they are a common accessory for women [19]. Based on the idea that society views women as sexual objects, pressure to dress in a sexualized manner may also be a daily manifestation of self-objectification.

To date, no quantitative survey measures have been developed to assist researchers in assessing the pressure women feel to use makeup and wear sexualized clothing. Thus, the purpose of the current set of studies was to create two questionnaires examining behaviors and attitudes associated with makeup and sexualized clothing use. In Study 1, the goal was to create measures assessing the pressure women feel to wear makeup and sexualized clothing; the goal of Study 2 was to confirm the factor structures from Study 1 and assess the incremental validity of the scales.

### Study 1

In Study 1, we developed items designed to assess beliefs and practices related to wearing makeup and sexualized clothing. We used an exploratory factor analysis (EFA) to examine the factor structure of the items. To further investigate the construct validity of the developed questionnaires, we hypothesized that the scales for makeup and sexualized clothing would be positively associated with self-objectification. Previous research indicates that self-objectification is concurrently associated with body dissatisfaction, eating disorder pathology, and thin-ideal internalization [20, 21]; thus, we hypothesized that these variables would be positively associated with scores on the makeup and sexualized clothing scales. Specifically, we expected that both a) greater discomfort when not wearing makeup and b) greater pressure to dress in sexualized clothing would be associated with greater body dissatisfaction, thin-ideal internalization, and preoccupations with shape and weight. Support for these hypotheses would corroborate the construct validity of the new questionnaires.

## Method

### Participants and procedure

All first-year undergraduate women residing on all campuses of a large, public university in the southwestern U.S. received an email asking them to fill out an online health behavior survey (*N* = 5284). In the email, women 18 years of age or older were invited to participate. This study was approved by the University’s Institutional Review Board and women completed an online consent form prior to completing the study. The online consent form, further re-affirmed that participants had to be 18 years of age. For this study, a total of 403 first-year college women (8%) participated in the online survey. Participants received $15 for their participation. Participants ranged in age from 18 to 32 years old (*M*_age_ = 18.79; *SD* = 1.14). The ethnic breakdown of the sample was Hispanic (22%), Non-Hispanic (71%), and missing data (8%). The majority of participants identified as Caucasian (65%) with the remaining identifying as Asian (14%), African American (8%), Pacific Islander/Hawaiian (2%), Native American/Alaskan (3%), and Other (3%). Participants were able to endorse more than one race. In addition to completing the newly created Makeup and Sexualized Clothing Questionnaires, participants completed validated measures of body satisfaction, eating disorder symptoms, self-objectification, and thin-ideal internalization.

### Item development

Body image experts collaborating at two universities in the southwestern U.S. (Trinity University and Arizona State University) created items based on direct experience running the Body Project, a body acceptance program, as well as comments from college women who participated in the program. Consistently, authors MP and CB noted women participating in the Body Project groups would discuss the use of makeup and sexualized clothing as daily costs to the ultra-thin ideal of beauty within the U.S. Initially, the authors created a total of 24 items to assess how frequently women did not wear makeup in work/academic settings, around the home, and while running errands such as grocery shopping. Also, items were developed concerning how attractive and competent women felt without makeup. Additionally, researchers developed items designed to measure the pressure women felt to dress in a sexualized way and how body image concerns impacted this pressure. The 24 items were discussed by 3 focus groups with a total of 27 undergraduate women who had previously participated in the Body Project. They discussed each item, the importance of the construct, and the wording of each item. Based on the focus group feedback, 13 items were retained and extensively edited. Then, items were reviewed by Dr. Eric Stice, the original creator of the Body Project.

The final items for the Makeup Questionnaire and the Sexualized Clothing Questionnaire assess women’s beliefs and attitudes regarding their attractiveness and confidence without makeup, their willingness to not wear makeup, and the pressure they perceive to wear sexualized clothing in light of how body image may impact this choice. To fill out the questionnaires, women responded to different statements assessed on a Likert scale ranging from 1 (*strongly disagree*) to 5 (*strongly agree*). For a complete list of the 13 items selected see Tables 1 and 2. Items 1 and 4 of the MUQ were reverse scored. Then, total scores were calculated for each scale by summing responses to the items, where higher scores indicate a greater discomfort without makeup and more pressure to wear sexualized clothing.

**Table 1:** Summary of Exploratory Factor Analysis Results for Makeup Questionnaire using Maximum Likelihood Estimation

	Factor Loadings	
Item	MUQ Unconfident	MUQ Unease
MUQ3. If I do not have makeup on, I feel less attractive.	**.87**	.03
MUQ6. Makeup makes me more attractive.	**.87**	−.18
MUQ5. If I do not have makeup on, I feel less competent.	**.54**	.31
MUQ4. I feel comfortable not wearing makeup to run errands (e.g., grocery store).	.23	**−.85**
MUQ1. I feel comfortable not wearing makeup to school.	−.11	**−.74**
MUQ2. I do not leave the house without any makeup on.	.25	**.54**
MUQ7. I believe women who are not wearing makeup are less attractive.	.21	.24
Eigenvalues	3.49	1.15
% of variance	49.85	16.45

Promax rotation was applied. Factor loadings in bold reflect items retained. *N* = 403

**Table 2:** Summary of Exploratory Factor Analysis Results for Sexualized Clothing Questionnaire using Maximum Likelihood Estimation

	Factor Loadings	
Item	SCQ Body Discomfort	SCQ Pressure
SCQ2: I do not wear revealing clothing because of body image concerns.	**1.00**	−.08
SCQ1: I purposely wear clothing that is less sexualized because of body image concerns.	**.80**	−.01
SCQ3: I feel pressure by society to dress in a sexualized manner.	.01	**.83**
SCQ4: On campus, I feel added pressure to dress in a sexualized manner.	.14	**.79**
SCQ5: Over time, I have noticed that my attire is becoming less conservative.	−.11	**.59**
SCQ6: The way women dress on campus bothers me.	.38	.11
Eigenvalues	2.85	1.36
% of variance	47.48	22.58

Promax rotation was applied. Factor loadings in bold reflect items retained. *N* = 403

### Body dissatisfaction

The Body Parts Satisfaction Scale-Revised (BPSS-R) assessed satisfaction with eleven body parts including the arms and stomach, as well as satisfaction with height, weight, and overall muscle tone [22]. Responses were recorded using a 6-point Likert scale ranging from 1 (*extremely dissatisfied*) to 6 (*extremely satisfied*), with an additional item assessing overall body satisfaction. For this measure, item scores were averaged, and higher scores indicated less pathology such that women with higher scores were more satisfied with their bodies [22]. For the current study, Cronbach’s alpha was .92.

### Shape and weight concern

Participants also completed the Eating Disorders Examination-Questionnaire (EDE-Q) Shape and Weight Concern subscales because these constructs are viewed as core psychopathology in bulimia nervosa [23]. These 13 items assessed how preoccupied women are with their current weight, as well as the shape of their body. The scores were averaged, and higher scores indicated greater pathology. Past research documents the internal consistency of the Shape Concern subscale; Cronbach’s alpha was .93 at a baseline assessment and .92 at the 12 to 16-week follow-up, with a strong test-retest reliability coefficient of .94. Scores on the Weight Concern subscale also display strong internal consistency with a Cronbach’s alpha was .89 at a baseline assessment and .89 at the 12 to 16-week follow-up, as with a strong test-retest reliability of .92 [23]. For this study, Cronbach’s alphas were .90 and .83 for Shape Concern and Weight Concern, respectively.

### Self-objectification

The Objectified Body Consciousness Scale (OBCS) Body Surveillance subscale comprises 8 items that assessed women’s preoccupation with their bodies, as well as how often they compared themselves to other women (e.g., “I often worry about whether the clothes I am wearing make me look good”) [24]. Participants responded using a 5-point Likert scale ranging from 1 (*strongly agree*) to 5 (*strongly disagree*). These scores were averaged, and a higher score indicated a high level of body surveillance. Cronbach’s alpha of Body Surveillance was .74.

### Thin-ideal internalization

Internalization of the idealized female body type (i.e., believing that thinner woman are more successful and happier in life) was assessed using two measures [25]. The first measure, Ideal-Body Stereotype Scale-Revised (IBSS-R), is a 10-item scale that asks women to report what they view as attractive, with responses recorded on a 5-point Likert scale ranging from 1 (*strongly disagree*) to 5 (*strongly agree*). Example item: “Slim women are more attractive” [25]. The scores were averaged, and greater scores indicated more thin-ideal internalization. Scores on the IBSS-R have demonstrated internal consistency with a Cronbach’s alpha of .91, test-retest reliability coefficient of .80, as well as predictive validity [25]. For the current study, Cronbach’s alpha of the IBSS-R was .90. The second measure used, the Body Image Culture Survey (BICS), includes 12 items assessing the extent to which a person believes that achieving the thin ideal would improve life [26]. Example item: “Being thinner than I am now would increase my sense of worth” [26]. Items were scored on a 5-point Likert scale ranging from 1 (*no chance*) to 5 (*certain to happen*), then scores were averaged with greater scores indicating more thin-ideal internalization. Cronbach’s alpha of the BICS was .97.

## Results

### Exploratory factor analysis of makeup questionnaire

Using SPSS 23, an exploratory factor analysis was conducted using maximum likelihood estimation. The Kiaser-Meyer-Olkin measure of sampling adequacy, which is the degree of common variance among the items, was .82, well above the recommended .70 [27]. The Bartlett’s Test of Sphericity was significant, χ^2^ (21) = 1090.08, *p* < .01. Using Kaiser’s [28] eigenvalue rule of thumb, two factors had eigenvalues greater than 1.0, which was confirmed further by visual inspection of a scree plot [29]. Based on the results of the factor analysis, two factors were retained. A promax rotation was applied because it was assumed the latent factors would be correlated. After rotation, the two factors accounted for 66.30% of the variance. Both factors were moderately correlated (*r* = .56). Out the 7 items, 3 loaded onto the first factor, and 3 loaded onto the second factor. See Table 1 for a summary of the factor loadings. Items loading highly on the first factor to emerge referred to feelings of unattractiveness and lack of competence that women felt when not wearing makeup and was labeled “Unconfident.” Items loading highly on the second factor to emerge referred to lack of comfort when not wearing makeup in daily situations and thus was labeled “Unease.” As item 7 failed to load onto any factor, it was eliminated from the final measure. Internal consistency for the scales was .82 for MUQ Unconfident, and .77 for MUQ Unease.

### Exploratory factor analysis of sexualized clothing questionnaire

A second exploratory factor analysis was conducted using maximum likelihood estimation to assess the items of the Sexualized Clothing Questionnaire (SCQ). The Kiaser-Meyer-Olkin Measure was .69 [27]. The Bartlett’s Test of Sphericity was significant, χ^2^ (15) = 938.66, *p* < .01. Using Kaiser’s [28] eigenvalue rule of thumb, two factors had eigenvalues greater than 1.0, which was further confirmed by visual inspection of a scree plot [29]. A factor analysis was repeated, and two factors were retained. A promax rotation was applied as it was theorized the latent factors would be correlated. After rotation, the two factors accounted for 70.06% of the variance, and the factors were moderately correlated (*r* = .44). Out of the 6 items, 2 loaded onto the first factor, and 3 loaded onto the second factor. Table 2 provides a summary of the factor loadings. Items loading highly on the first factor related to not dressing in a sexualized fashion due to body image concerns, labeled “Body Discomfort.” Items loading highly on the second factor related to societal pressure to wear sexualized clothing; as such, it was labeled “Pressure.” Item 6 was deleted due to low factor loadings and because the question focused on perceptions of others, while all the other questions on the questionnaire were focused on the self. Spearman-Brown analysis for SCQ Body Discomfort was .77, and internal consistency for SCQ Pressure was .78.

### Correlational analyses

Pearson correlations tested the associations between MUQ Unconfident, MUQ Unease, SCQ Body Discomfort, SCQ Pressure, and self-objectification and other hypothesized correlates including body satisfaction, shape and weight concerns, and thin-ideal internalization. All correlations were in the expected direction (see Table 3). Higher thin-ideal internalization and self-objectification scores were associated with higher scores on the MUQ and SCQ scales. Similarly, higher body satisfaction scores were associated with lower scores on the MUQ and SCQ scales. MUQ Unconfident, MUQ Unease, and SCQ Pressure were all significantly correlated with body satisfaction (i.e., BPSS-R), self-objectification (i.e., Body Surveillance), Shape Concern, Weight Concern, and both thin-ideal internalization scales (i.e., IBSS-R, BICS). Additionally, the Body Discomfort scale on the SCQ was significantly correlated with all measures except Body Surveillance. Overall, the newly created Makeup Questionnaire scales and the Sexualized Clothing Questionnaire scales were significantly correlated with measures commonly used to assess self-objectification and its consequences.

**Table 3:** Bivariate correlations between measures and descriptive statistics for study 1

	1	2	3	4	5	6	7	8	9	10
1. MUQ Unconfident										
2. MUQ Unease	.536^b^									
3. SCQ Body Discomfort	.234^b^	.151^b^								
4. SCQ Pressure	.256^b^	.067	.349^b^							
5. Thin-ideal Stereotype	.333^b^	.131^b^	.167^b^	.245^b^						
6. Body Image Culture	.322^b^	.148^b^	.456^b^	.267^b^	.352^b^					
7. Shape Concern	.237^b^	.129^a^	.448^b^	.236^b^	.323^b^	.539^b^				
8. Weight Concern	.258^b^	.142^b^	.384^b^	.214^b^	.288^b^	.527^b^	.770^b^			
9. Body Part Satisfaction	−.245^b^	−.155^b^	−.421^b^	−.232^b^	−.185^b^	−.461^b^	−.561^b^	−.499^b^		
10. Body Surveillance	.140^b^	.229^b^	.095	.134^b^	.157^b^	.103^a^	.159^b^	.146^b^	.179^b^	
Mean	9.244	6.821	5.133	8.216	3.734	32.77	2.420	2.248	60.104	24.839
Standard Deviation	3.247	3.270	2.292	2.950	0.775	13.202	0.967	0.983	16.002	3.759

*N* = 403. ^a^Correlation is significant at the 0.05 level (2-tailed). ^b^Correlation is significant at the 0.01 level (2-tailed). IBSSR – thin ideal internalization; BICS – belief that attaining thin ideal will improve life; Shape Concern – dissatisfaction with body shape; Weight Concern – dissatisfaction with weight; BPSSR – body part satisfaction; Body Surveillance – self-objectification

## Discussion

This first study aimed to extend the current literature through the creation of measures assessing makeup use and pressure to wear sexualized clothing. Overall, results provided evidence for two scales for each new questionnaire. The Makeup Questionnaire (MUQ) comprised a 3-item scale labeled Unconfident (i.e., women’s feelings of attractiveness and competency not wearing makeup) and a 3-item scale labeled Unease (i.e., women’s willingness and comfort with not wearing makeup to school, the store, or out of the house in general). The Sexualized Clothing Questionnaire (SCQ) comprised a 2-item scale labeled Body Discomfort (i.e., the extent to which body image concerns impact women’s clothing selection) and a 3-item scale labeled Pressure (i.e., the perceived pressure women feel to dress in a sexualized way).

To provide support for the construct validity of scores on these new scales, it was hypothesized that the scales would be correlated with self-objectification and other known correlates, such as thin-ideal internalization and body dissatisfaction. Results revealed that MUQ Unconfident, MUQ Unease, and SCQ Pressure were significantly correlated with self-objectification and its consequences in the expected direction (the IBSS-R, BICS, Shape Concern, Weight Concern, BPSS-R, and Body Surveillance). That is, higher self-objectification scores were associated with more discomfort without makeup, less confidence without makeup, and more pressure to dress in a sexualized manner. This supports self-objectification theory [5], which suggests that women who are more prone to self-objectify are more likely to experience appearance anxiety, manifested in the current study as less confidence without makeup and more pressure to dress sexually to fit a societal ideal. Interestingly, SCQ Body Discomfort was not significantly correlated with self-objectification, but was significantly correlated with thin-ideal internalization and all of the body dissatisfaction measures, suggesting that body discomfort regarding sexualized clothing may be more strongly related to body dissatisfaction.

The significant correlations within Table 3 range from .10 to .56. The lowest correlations tended to be with the Body Surveillance scale, a measure of self-objectification; however the correlations are consistent with the existing literature using the same measures [30, 31]. In addition, correlations among thin-ideal internalization, body parts satisfaction, shape concerns and weight concerns are representative of the existing literature [31, 32]. This suggests are sample was consistent with the literature on known questionnaires [30–32].

### Study 2

In Study 2, we aimed to replicate the results of Study 1, demonstrating that the items selected from Study 1 and factor structures replicate using a confirmatory factor analysis (CFA). Similar to Study 1, the scales were examined using correlational analyses with self-objectification and its consequences (e.g., body dissatisfaction, thin-ideal internalization, etc.). This study also examined the test-retest reliability of the scales. Additionally, we assessed the incremental validity of the created scales for each questionnaire using hierarchical multiple regression models. Incremental validity is important because the analysis reveals whether our created measures uniquely predict disordered behaviors of women, specifically self-objectification and shape and weight concerns, over and above the influence of known predictors. Previous research documents that thin-ideal internalization predicts self-objectification and body dissatisfaction, and self-objectification has been found to predict body dissatisfaction [21, 32]. Additionally, thin-ideal internalization and body dissatisfaction predict eating disorder pathology using longitudinal data [21, 32, 33]. Given that thin-ideal internalization and body dissatisfaction are closely intertwined with predicting self-objectification and eating disorder pathology, such as shape and weight concerns, we adjusted for thin-ideal internalization and body dissatisfaction in our analysis of incremental validity. Using longitudinal data, we hypothesized that our scales would uniquely predict changes in self-objectification and shape and weight concerns 12–16 weeks later (T2), while adjusting for thin-ideal internalization and body dissatisfaction at Time 1 (T1). Body Mass Index (BMI) was included as a covariate in these analyses because past research has found body dissatisfaction and body surveillance to be higher among overweight/obese women [30, 34].

### Method

#### Participants and procedure

All first-year undergraduate women residing on all campuses of a large, public university in the southwestern U.S. received an email asking them to fill out an online health behavior survey. For this study, a total of 174 first-year college women participated in the online survey. These participants were different from the women who participated in Study 1. Participants ranged in age from 18 to 23 years old (*M*_age =_ 18.46; *SD* = .76). The ethnic breakdown of the sample was Hispanic (27%), Non-Hispanic (71%), and missing data (2%). The majority of participants identified as Caucasian (76%) with the remaining 24% comprised of Asian (15%), African American (9%), Pacific Islander/Hawaiian (3%), Native American/Alaskan (1%), and Other (3%). Participants were able to endorse more than one race. This study was approved by the University’s Institutional Review Board and women completed an online consent form prior to completing the study. Participants received $15 for their participation. Inclusion criteria consisted of being an undergraduate woman residing on campus and a minimum of 18 years of age. Women agreed to fill out a baseline online survey (T1) and a follow-up online survey 12 to 16 weeks later (T2).

### Measures

#### Makeup use

The MUQ developed in Study 1 was found to have two scales: Unconfident, which assessed how wearing makeup impacts women’s feelings of competency and attractiveness, and Unease, which assessed how willing women are to not wear makeup in public places. Each scale consisted of 3 items rated on a Likert scale from 1 (*strongly disagree*) to 5 (*strongly agree*), where higher scores indicated less confidence and comfort while wearing makeup. Cronbach’s alpha of MUQ Unconfident was .77 for baseline and .76 for T2. Cronbach’s alpha of MUQ Unease was .80 for baseline and .82 for T2.

#### Use of sexualized clothing

The SCQ developed in Study 1 was found to have two scales: Body Discomfort, consisting of 2 items that assessed how body image concerns impact what women wear, and Pressure, consisting of 3 items that assessed how pressured women feel to dress in a sexualized way. Each item was rated on a Likert scale from 1 (*strongly disagree*) to 5 (*strongly agree*), where higher scores indicated more body discomfort and pressure with regards to wearing sexualized clothing. Internal consistency of the SCQ Body Discomfort was assessed using a Spearman-Brown analysis and was .83 for baseline and .78 for T2. Cronbach’s alpha of SCQ Pressure was .79 for baseline and .82 for T2. For more detailed information on additional measures, see the descriptions in Study 1.

#### Body image scales

Similar to Study 1, a number of well-known body image self-report questionnaires were used at both time points. Cronbach’s alpha of the BPSS-R [22]was .88 for baseline and .92 for T2. Cronbach’s alpha of Shape Concern [23] was .87 for baseline and .90 for T2. Cronbach’s alpha of Weight Concern [23] was .79 for baseline and .78 for T2. Cronbach’s alpha of Body Surveillance [24] was .78 for baseline and .73 for T2. Cronbach’s alpha of the IBSS-R [25] was .83 for baseline and .88 for T2. Cronbach’s alpha of the BICS [26] was .97 for baseline and .97 for T2.

#### Body mass index (BMI)

Participants self-reported their height and weight. BMI was calculated using the United States Centers for Disease Control’s gender- and age-based charts for adults [35]. BMI was included as a covariate for the incremental validity analyses.

### Results

After deletion of participants with incomplete data, the remaining 153 participants provided complete data on the questionnaires across both time points.

#### Confirmatory factor analysis of makeup questionnaire

Using Mplus [36], confirmatory factor analysis was conducted using baseline scores. For the Makeup Questionnaire, fit of a two-factor model was evaluated following the EFA findings in Study 1. We used maximum likelihood estimation and allowed Factors 1 and 2 to covary [37]. According to several fit indices, the two-factor model was an adequate fit, χ^2^ (8) = 15.48, *p* > .05 [38] CFI was .98 and above the recommended .95 [39], AGFI was .91 and above the recommended .80 [40], RMSEA was .08, which is considered moderate fit [41], and PClose was .19 and above the recommended .05 [40].

#### Confirmatory factor analysis of sexualized clothing questionnaire

A second CFA was conducted using only baseline data for the Sexualized Clothing Questionnaire. Using a similar strategy as above, the model was an adequate fit, χ^2^ (4) = 5.93, *p* > .05 [38], CFI was .99 and above the recommended .95 [39], AGFI was .94 and above the recommended .80 [40], RMSEA was .06, which is considered good fit [41], and PClose was .37 and above the recommended .05 [40]. Upon closer analysis, Item 5 was conceptually different than the rest of the items on the measure. All the other items in the SCQ measure current feelings, while Item 5 assesses a retrospective inspection of attire over time. In addition, based on the standardized estimates of the item loadings, item 5 had a low loading of .37. For these reasons, it was deleted from the model. After the item was deleted, a two-factor model provided an excellent fit, χ^2^ (1) = .03, *p* > .05 [38], CFI was 1.00 [39], AGFI was .99 [40], RMSEA was .00 [41], and PClose was .89 [40].

#### Test-retest reliability

MUQ Unconfident scale scores had strong temporal stability across 12–16 weeks, *r*(151) = .68, *p* < .01 [39]. MUQ Unease scores also displayed strong temporal stability, *r*(151) = .72, *p* < .01 [39]. Similarly, SCQ Body Discomfort scores had strong temporal stability, *r*(151) = .69, *p* < .01 [39]. SCQ Pressure scores displayed moderate stability, *r*(151) = .51, *p* < .01 [39].

#### Correlational analyses

Pearson correlations were used to test associations between MUQ Unconfident, MUQ Unease, SCQ Body Discomfort, SCQ Pressure, and the Objectified Body Consciousness Scale, as well as measures examining the consequences of self-objectification at T1 and T2. The cross-sectional correlations for MUQ Unconfident at T1 were similar to the results from Study 1. MUQ Unconfident was significantly correlated with self-objectification, body dissatisfaction, shape and weight concerns, and thin-ideal internalization. MUQ Unease was previously significantly correlated with body dissatisfaction, eating pathology, self-objectification, thin-ideal internalization in the expected direction in Study 1. When tested with the current sample at T1, the cross-sectional correlations between the MUQ Unease scale and all the other variables were significant and in the expected direction, except the correlations with weight and shape concerns were not significant (see Table 4). In other words, as MUQ Unease increased, scores on thin-ideal internalization and self-objectification increased, where scores on body satisfaction decreased. SCQ Pressure was previously correlated with self-objectification, thin-ideal internalization, and body dissatisfaction in the expected direction in Study 1, but in the present sample this scale was not correlated with body dissatisfaction. The cross-sectional correlations between SCQ Body Discomfort with self-objectification, body dissatisfaction, thin-ideal internalization, and shape and weight concerns were significant, while in Study 1 SCQ Body Discomfort was not correlated with self-objectification. All cross-sectional correlations at T2 were in the expected direction: women who scored higher on thin-ideal internalization and self-objectification tended to score higher on MUQ and SCQ scales, and women who scored lower on body satisfaction tended to score higher on the MUQ and SCQ scales.

**Table 4:** Study 2 Cross-sectional, Bivariate Correlations between Measures for Time 1 and Time 2

		1	2	3	4	5	6	7	8	9	10
Time 1											
1. MUQ Unconfident		--	.551^b^	.202^a^	.128	.283^b^	.312^b^	.186^a^	.186^a^	−.306^b^	.374^b^
2. MUQ Unease		.510^b^	--	.217^b^	.084	.220^b^	.220^b^	.152	.130	−.193^a^	.398^b^
3. SCQ Body Discomfort		.220^b^	.144	--	.256^b^	.203^a^	.388^b^	.506^b^	.498^b^	−.417^b^	.332^b^
4. SCQ Pressure		.045	−.052	.257^b^	--	.374^b^	.212^b^	.173^a^	.230^b^	−.088	.257^b^
5. Thin-ideal Stereotype		.335^b^	.092	.223^b^	.211^b^	--	.385^b^	.260^b^	.274^b^	−.117	.382^b^
6. Body Image Culture		.403^b^	.159^a^	.529^b^	.132	.389^b^	--	.645^b^	.699^b^	−.416^b^	.394^b^
7. Shape Concern		.249^b^	.037	.620^b^	.271^b^	.380^b^	.649^b^	--	.847^b^	−.608^b^	.503^b^
8. Weight Concern		.300^b^	.081	.591^b^	.243^b^	.390^b^	.746^b^	.833^b^	--	−.555^b^	.391^b^
9. Body Part Satisfaction		−.289^b^	−.153	−.500^b^	−.180^a^	−.311^b^	−.538^b^	−.592^b^	−.609^b^	--	−.405^b^
10. Body Surveillance		.412^b^	.425^b^	.297^b^	.185^a^	.397^b^	.435^b^	.453^b^	.471^b^	−.460^b^	--
Time 2											
Mean	Time 1	9.961	7.255	5.307	5.516	3.727	32.536	2.426	2.230	59.665	27.654
	Time 2	9.915	7.196	5.477	5.647	3.738	34.314	2.431	2.173	59.158	27.608
Standard Deviation	Time 1	2.967	3.176	2.396	2.081	0.610	12.541	0.885	0.864	14.650	4.772
	Time 2	2.707	3.169	2.227	2.150	0.662	13.599	0.904	0.881	17.054	4.487

^a^Correlation is significant at the 0.05 level (2-tailed). ^b^Correlation is significant at the 0.01 level (2-tailed). *N* = 153

Bivariate correlations for time 1 are presented above the diagonal and bivariate correlations for time 2 are presented below the diagonal

MUQ Unease was not significantly correlated with body dissatisfaction, shape and weight concerns, and one measure of thin-ideal internalization (IBSS-R; see Table 4). In contrast, scores on the SCQ Pressure scale were not significantly associated with scores on the BICS. Overall, MUQ Unconfident and SCQ Body Discomfort supported the hypothesis for Study 2 because they were both significantly correlated with the measures assessing body dissatisfaction, shape and weight concerns, self-objectification, and thin-ideal internalization at two different points in time. SCQ Pressure was significantly correlated with the majority of these measures. However, MUQ Unease only correlated with one scale measuring thin-ideal internalization and another scale measuring self-objectification at T2 (BICS and Body Surveillance), which differed from what was found at T1, and at Study 1.

#### Incremental validity analyses

The incremental validity of the scales from the MUQ and the SCQ was assessed using a series of hierarchical multiple regression models to predict changes in self-objectification, shape concern, and weight concern after adjusting for known predictors of these outcomes. This was done to assess whether daily consequences of self-objectification (e.g., wearing makeup and sexualized clothing) could predict changes in self-objectification as well as shape and weight concerns (i.e., core psychopathology for bulimia nervosa). Therefore, it was hypothesized that these scales would uniquely predict changes in self-objectification and shape and weight concerns, while adjusting for common predictors of self-objectification and eating disorder pathology (e.g., thin-ideal internalization, body dissatisfaction).

Step 1 was a two-occasion autoregressive model, in which scores on the outcome measure (e.g., self-objectification) at T1 were added to predict the same outcome at T2. Then, in Step 2, scale scores from the MUQ and SCQ at T1 were added (in separate models). In Step 3, scale scores at T2 were added to assess whether *changes* in the new measures of interest were associated with changes in each of the three outcomes. Lastly, in Step 4, common predictors of the outcomes (e.g., thin ideal internalization, body dissatisfaction) were added to the models to assess whether the new measures contributed unique variance in predicting the outcomes (see Table 5). These analyses were conducted in SAS 9.3.

**Table 5:** Summary of significant hierarchical multiple regression models predicting self-objectification, shape concern, and weight concern

	B	*SE* B	β	*t*	*R* ^2^	*F*
Predicting T2 self-objectification						
T1 self-objectification	0.61	0.06	0.65	9.48**		
T1 MUQ Unease	−0.26	0.12	−0.19	−2.25*		
T2 MUQ Unease	0.48	0.11	0.34	4.28**		
Thin-ideal internalization	−0.26	0.43	−0.04	−0.61		
Body dissatisfaction	−0.03	0.02	−0.10	−1.68	0.56	37.83**
Predicting T2 shape concern						
Model 1						
T1 Shape Concern	0.47	0.08	0.46	6.18**		
T1 SCQ Body Discomfort	0.0004	0.03	0.001	0.01		
T2 SCQ Body Discomfort	0.13	0.03	0.33	4.19**		
Thin-ideal internalization	0.06	0.08	0.04	0.68		
Body dissatisfaction	−0.01	0.004	−0.10	−1.45	0.57	38.71**
Model 2						
T1 Shape Concern	0.60	0.08	0.58	7.87**		
T1 SCQ Pressure	−0.03	0.03	−0.08	−1.12		
T2 SCQ Pressure	0.08	0.03	0.19	2.83**		
Thin-ideal internalization	0.07	0.09	0.05	0.80		
Body dissatisfaction	−0.01	0.005	−0.15	−2.10*	0.51	31.50**
Predicting T2 weight concern						
Model 1						
T1 Weight Concern	0.47	0.07	0.46	6.73**		
T1 SCQ Body Discomfort	0.01	0.03	0.03	0.34		
T2 SCQ Body Discomfort	0.11	0.03	0.28	3.70**		
Thin-ideal internalization	0.07	0.08	0.05	0.85		
Body dissatisfaction	−0.01	0.004	−0.17	−2.59*	0.60	44.03**
Model 2						
T1 Weight Concern	0.57	0.07	0.57	8.20**		
T1 SCQ Pressure	−0.03	0.03	−0.08	−1.10		
T2 SCQ Pressure	0.06	0.03	0.14	2.22*		
Thin-ideal internalization	0.09	0.09	0.07	1.06		
Body dissatisfaction	−0.01	0.004	−0.23	−3.44**	0.55	36.25**

*N* = 157. Thin-ideal internalization and body dissatisfaction were measured at T1. B = unstandardized partial regression coefficient. *SE* B = standard error of the unstandardized estimate. β = standardized beta weight. Values for R^2^ are adjusted. Scale scores not presented here were not significant predictors of any of these three outcomes (*p*s > .08). Models were highly similarly when also controlling for BMI. *p < .05. **p < .01

##### Self-objectification

In the first set of models, MUQ Unconfident, MUQ Unease, SCQ Body Discomfort, and SCQ Pressure were used to predict self-objectification. Changes in MUQ Unconfident from T1 to T2 did not significantly predict changes in self-objectification (*B* = .23, *p* = .10) above and beyond thin-ideal internalization and body dissatisfaction. However, as displayed in Table 5, increases in MUQ Unease from T1 to T2 were significantly associated with increases in self-objectification (*p* < .05). Changes in scores on MUQ Unease accounted for approximately 5% of the variance in self-objectification at T2 (Δ*R* ^2^ = .05) over and above other predictors, including the autoregressive component of the model (i.e., self-objectification predicting itself over time). Changes in SCQ Body Discomfort and SCQ Pressure did not significantly predict changes in self-objectification (*p*s > .10) above and beyond thin-ideal internalization and body dissatisfaction*.* Model results were highly similar when also adjusting for BMI.

##### Shape and weight concern

In the second set of models, Shape Concern and Weight Concern were the outcomes; MUQ Unconfident, MUQ Unease, SCQ Body Discomfort, and SCQ Pressure were used as the predictors. Changes in MUQ Unconfident and MUQ Unease did not significantly predict changes in Shape and Weight Concern (*p*s > .08) above and beyond thin-ideal internalization and body dissatisfaction. However, as displayed in Table 5, increases in SCQ Body Discomfort from T1 to T2 significantly predicted increases in Shape Concern and Weight Concern, while controlling for thin-ideal internalization and body dissatisfaction. Changes in scores on SCQ Body Discomfort accounted for approximately 7% and 6% of the variances in shape and weight concern, respectively, over and above other predictors. Additionally, as displayed in Table 5, increases in SCQ Pressure from T1 to T2 significantly predicted increases in Shape Concern and Weight Concern, while adjusting for thin-ideal internalization and body dissatisfaction. Changes in scores on SCQ Pressure accounted for approximately 2% and 1% of the variances in Shape and Weight Concern, respectively, over and above other predictors. Model results were highly similar when also adjusting for BMI.

## Discussion

The aim of Study 2 was to replicate the initial factor structure of the MUQ and SCQ found in Study 1. In addition, Study 2 aimed to explore and establish the psychometric properties of these scales. Overall, results from this study replicated the initial factor structure and items of the MUQ and SCQ found in Study 1. The correlational analyses of the MUQ Unease, SCQ Body Discomfort, and SCQ Pressure scales provided different results from Study 1, but the correlational analyses of MUQ Unconfident were similar to the results from Study 1. MUQ Unconfident and SCQ Body Discomfort supported the hypothesis that the makeup and sexualized clothing scales would be correlated with self-objectification and its consequences across both time points.

These results suggest the daily consequences of confidence regarding makeup use and body discomfort regarding wearing sexualized clothing are related to self-objectification, thin-ideal internalization, body dissatisfaction, and shape and weight concerns. SCQ Pressure was significantly correlated with the majority of the scales assessing self-objectification and its consequences. However, MUQ Unease only correlated with thin-ideal internalization and self-objectification at T2. In total, there were only 5 significant correlations from Study 1 that did not replicate in Time 1 of Study 2. The different correlational results in Study 2 might suggest that wearing makeup and sexualized clothing are impacted by changes over time and/or across different groups of college women. More specifically, one group of college women may have lost interest in trying to achieve the unrealistic beauty expectation of society over time, where another group may have felt an increase in pressure to wear makeup and sexualized clothing.

Results from hierarchical multiple regression analyses indicated that changes in scores on the MUQ were associated with changes in self-objectification, whereas changes in scores on the SCQ were related more to changes in shape concern and weight concern. These results support our hypothesis. Specifically, changes in MUQ Unease significantly predicted changes in self-objectification, even when controlling for initial scores on thin-ideal internalization and body dissatisfaction. On the other hand, changes in SCQ Body Discomfort and SCQ Pressure significantly predicted changes in shape concern and weight concern, while adjusting for thin-ideal internalization and body dissatisfaction. However, it is important to note that the SCQ scales uniquely predicted a small amount of variance in shape and weight concerns. Overall, the incremental validity analysis revealed that our scales offer unique prediction of self-objectification and eating disorder symptoms over and above commonly used measures. Specifically, the daily action of wearing makeup can predict changes in levels of self-objectification while controlling for common predictors of self-objectification (i.e., thin-ideal internalization and body dissatisfaction). These results relate to self-objectification theory in that wearing makeup could be an indication of women viewing themselves as an object. Additionally, the daily action of wearing sexualized clothing can predict changes in shape and weight concerns while controlling for common predictors of eating disorder pathology (i.e., thin-ideal internalization and body dissatisfaction).

Despite the longitudinal design, it is possible that changes in self-objectification or shape and weight concerns prompt changes in makeup or sexualized clothing use. As such, cause and effect should not be inferred from the present analyses. It is also possible that these processes are reciprocally linked over time, or a third variable could be producing change in scores on both the new measures and the outcomes of interest.

### General discussion

The overall goal of these studies was to create a valid and reliable measure assessing women’s comfort level wearing or not wearing makeup and the pressure women feel to dress in a sexualized way. These measures were created in Study 1, and an EFA was used to better understand whether there are underlying components of these constructs. Two scales emerged from the EFA of the Makeup Questionnaire. Factor 1 was labeled Unconfident because items assessed how confident women feel wearing or not wearing makeup, and Factor 2 was labeled Unease because items assessed how comfortable women feel wearing or not wearing makeup to the store, school, or out of the house in general. Two scales emerged from the EFA of the Sexualized Clothing Questionnaire. Factor 1 was labeled Body Discomfort because items assessed if body image concerns prevent women from wearing sexualized clothing, and Factor 2 was labeled Pressure because items assessed the pressure women feel to dress in a sexualized way. Pearson correlations between MUQ Unconfident, MUQ Unease, SCQ Body Discomfort, SCQ Pressure, and self-objectification and other correlates revealed that the MUQ and SCQ scales significantly correlate with self-objectification, thin-ideal internalization, body dissatisfaction, and eating disorder symptoms. All correlations were in the expected direction: women who scored higher on thin-ideal internalization and self-objectification tended to score higher on MUQ and SCQ scales, and women who scored lower on body satisfaction tended to score higher on the MUQ and SCQ scales.

A second study confirmed the factor structure from Study 1 using a CFA of each scale and indicated that these scales were relatively stable over time. The CFA of SCQ Body Discomfort confirmed the factor structure found in Study 1, and this scale was found to be stable over time. However, the CFA of SCQ Pressure deleted an item from the original scale, and this scale had the potential to change over time. After the CFA conducted in Study 2, the final scales were: the 3-item Unconfident scale, the 3-item Unease scale, the 2-item Body Discomfort scale, and the 2-item Pressure scale. Overall, confidence, comfort, and body discomfort were stable over time according to the test-retest reliability scores. Correlational analyses revealed differences from Study 1, suggesting that wearing makeup and sexualized clothing may be impacted by changes over time across different groups of people. Additionally, the incremental validity of the scales was assessed using a series of hierarchical multiple regression models to predict changes in self-objectification, shape concern, and weight concern after adjusting for known predictors of these outcomes. Results supported the hypotheses that MUQ Unconfident and MUQ Unease would predict changes in self-objectification. While SCQ Body Discomfort and SCQ Pressure predicted changes in shape concern and weight concern, the amount of unique variance accounted for was rather small and potentially not clinically meaningful.

This study contributes to the current research on makeup use and sexualized clothing, as it provides researchers and clinicians a way to measure the pressure women feel to wear makeup and dress in a sexualized way. It is important to note that across the three assessment occasions women reported moderate levels of makeup and sexualized clothing use, with approximately 25% of the women across all occasions reporting high levels of appearance anxiety and pressure. This suggests that makeup wearing and pressure to dress in a sexualized manner are salient in their daily lives, and thus important constructs to assess in women. Given that MUQ significantly predicted changes in self-objectification, the MUQ may measure daily behavior women engage in that encourages self-objectification (or possibly results from self-objectification). This is a unique contribution to self-objectification theory; previous studies have focused on trait levels of appearance anxiety and body dissatisfaction, whereas MUQ is an assessment of a daily behavior.

This study also contributes to the study of body dissatisfaction. Body dissatisfaction is most frequently measured in the literature using trait-level measures [42]. The SCQ was designed as a more daily consequence of thin ideal internalization and body dissatisfaction. Our results across Study 1 and 2 support this. SCQ was consistently associated with body dissatisfaction. In addition, SCQ at baseline predicted increases in Shape and Weight Concerns scales at T2. The sexualized clothing scales allow researchers to measure a part of this sexual objectification theory because the scales assessed how pressured women felt to dress in a sexualized way, as well as if the weight and shape of their body prevented them from wearing sexualized clothing. Therefore, the sexualized clothing scales help determine the degree to which a woman is separating her clothing from who she is as a person.

There are also a few limitations of this study. The first limitation is the sample comprising exclusively college women, perhaps limiting whether these results generalize to older women or young adult women not attending college. Secondly, self-report measures, like the questionnaires, are prone to certain types of biases that could impact the results. Although self-report measures allow women to admit to feelings and behaviors that they may be reluctant to reveal in an interview setting, participants are unable to expand on and explain their feelings about makeup and sexualized clothing in greater depth. Another limitation to this present study is time, as this study was conducted under a limited time frame of three to four months between time 1 and time 2; more assessment time points across a longer time frame would have allowed for more sophisticated analysis of how makeup and sexualized clothing related to self-objectification.

## Conclusions

Despite these limitations, this study calls attention to the pressure women feel to wear makeup and sexualized clothing. Additionally, this study reveals a need for our society to develop more sensitive measures to assess this pressure, so that we can develop better ways to help women resist this pressure to wear makeup and sexualized clothing. The MUQ scales give researchers a way to measure and record the pressure women feel to conform to society’s ideal image of beauty through wearing facial makeup. Additionally, the SCQ scales provide researchers with a tool to assess the impact of society’s views regarding sexualization and sexualized clothing on young women. These new measures assessing the pressure women feel to wear makeup and sexualized clothing make people aware of this pressure, which is the first step toward addressing this pressure.
